# Fundamentals of aerosol therapy in critical care

**DOI:** 10.1186/s13054-016-1448-5

**Published:** 2016-10-07

**Authors:** Jayesh Dhanani, John F. Fraser, Hak-Kim Chan, Jordi Rello, Jeremy Cohen, Jason A. Roberts

**Affiliations:** 1 Burns, Trauma and Critical Care Research Centre, The University of Queensland, Brisbane, Australia; 2 Department of Intensive Care Medicine, Royal Brisbane and Women’s Hospital, Level 3, Ned Hanlon Building, Herston, 4029 QLD Australia; 3 Department of Intensive Care Medicine, The Prince Charles Hospital, Brisbane, Australia; 4 Critical Care Research Group, The University of Queensland, Brisbane, Australia; 5 Advanced Drug Delivery Group, Faculty of Pharmacy, The University of Sydney, Sydney, NSW Australia; 6 Pharmacy Department, Royal Brisbane and Women’s Hospital, Herston, Brisbane, Australia; 7 School of Pharmacy, The University of Queensland, Brisbane, Australia; 8 Critical Care Department, Hospital Vall d’Hebron, Barcelona, Spain; 9 CIBERES, Vall d’Hebron Institut of Research, Barcelona, Spain; 10 Department of Medicine, Universitat Autonoma de Barcelona, Barcelona, Spain

## Abstract

Drug dosing in critically ill patients is challenging due to the altered drug pharmacokinetics–pharmacodynamics associated with systemic therapies. For many drug therapies, there is potential to use the respiratory system as an alternative route for drug delivery. Aerosol drug delivery can provide many advantages over conventional therapy. Given that respiratory diseases are the commonest causes of critical illness, use of aerosol therapy to provide high local drug concentrations with minimal systemic side effects makes this route an attractive option. To date, limited evidence has restricted its wider application. The efficacy of aerosol drug therapy depends on drug-related factors (particle size, molecular weight), device factors, patient-related factors (airway anatomy, inhalation patterns) and mechanical ventilation-related factors (humidification, airway). This review identifies the relevant factors which require attention for optimization of aerosol drug delivery that can achieve better drug concentrations at the target sites and potentially improve clinical outcomes.

## Background

The main goal of aerosolization is to achieve high drug concentrations in lung tissue. Aerosol therapy has been used as part of the treatment for a variety of respiratory diseases [1]. Indeed, there is also significant interest in the utilization of the respiratory system as a portal for systemic therapy [2] of conditions that are not purely respiratory in nature. Factors such as a large surface area, thin air–blood barrier and vascular epithelium coupled with low first-pass metabolism and enzymatic activity could achieve high bioavailability for aerosolized drug therapy [3]. The possibility of achieving very high local drug concentrations at the therapeutic site for respiratory pathology, rapid onset of action and lower systemic side effects [4] has thus led to a renewed interest in the field of aerosolized drug therapy in intensive care.

Datura administration in India, tobacco in ancient South America and smoking pipes from North American Indians are some of the early uses of airways as a route for systemic drug delivery [5, 6]. Vaporized opium was used as a treatment for cough. Anticholinergic properties of inhaled herbal preparations were used to treat asthma and inhaled epinephrine was first used around 1910 [7]. Aerosolized therapy is used for many therapies now including bronchodilators and corticosteroids, with a particular interest in antibiotic administration re-emerging recently. Although there are references to the use of inhaled penicillin as early as 1946 [5], the first randomized controlled trial of inhaled antibiotics was first reported in cystic fibrosis (CF) patients in 1981. In critical care, endotracheal antibiotic administration was first reported in the 1970s [8], when Klastersky et al. reported that endotracheal polymyxins were effective for prevention of ventilator-associated pneumonia in tracheostomized patients [9–11]. Following these and other studies it was noted that there were adverse effects such as bronchospasm and poor tolerance [9] as well as concerns regarding emergence of drug resistance associated with prolonged (>3 weeks) endotracheal administration and pharyngeal aerosolization [12]. This led to a reduction in the use of inhaled antibiotics. Even so, some investigators continued to prescribe intratracheal antibiotics in the critically ill patient, often successfully, especially in drug-resistant pneumonias [13, 14]. Antibiotic instillation practices were used in some early studies, but this practice was largely abandoned in the 1980s. Subsequent use of bench models enabled an improved understanding of the aerosolization factors such as optimal ventilator parameters, device position in the circuit and effects of humidity to enable optimal therapy [15–17]. This work was then supplemented with antibiotic studies in experimental pneumonia that demonstrated higher lung tissue concentrations of antibiotics [18]. The later development of ‘new generation’ devices such as the ultrasonic nebulizer and the vibrating mesh nebulizer (VMN) encouraged further study and application of aerosol therapy in critical care because of the ability of these devices to consistently generate desired aerosol particle sizes which are considered optimal for deep lung penetration [17, 19, 20].

Previously, the formulation of drugs used for aerosolization was the reconstituted form of compounds developed for parenteral administration. These were poorly tolerated by patients due to hyperosmolarity and added preservatives (i.e. phenols), which induced bronchial irritation and bronchospasm, leading to abandonment of this route of therapy. These formulation issues were particularly problematic for antibiotics until the 1990s, when aerosolized tobramycin was evaluated in patients with CF chronically infected with increasingly resistant *Pseudomonas aeruginosa* [21, 22]. A number of high-quality studies using preservative-free and iso-osmolar formulations of tobramycin showed improvements in lung function, a decreased exacerbation rate and reductions in sputum bacterial load [21–23]. These results have encouraged further developments in the application of aerosolized antibiotics in non-CF patient populations such as critical care. In the critically ill patient, certain anatomico-physiological changes can significantly affect the pharmacokinetics (PK)–pharmacodynamics (PD) characteristics, thus causing dosing difficulties [24]. Mechanically ventilated patients pose a challenge for the effective delivery of aerosolized drugs [25]. These various factors need to be considered and optimized to achieve desired therapeutic outcomes with aerosolized drug therapy [25].

The research interest in aerosol drug therapy in critically ill patients is not yet reflected in the bench-to-bedside transfer of knowledge. One report mentions that up to 95 % of intensivists are routinely prescribing aerosol medications [26]. This report also highlighted the lack of application of scientific principles during therapy and indicated the need for education and research in the bench-to-bedside transfer of knowledge [26]. In another study, every fourth critically ill patient and every fifth ventilated patient received aerosol therapy [27]. A recent International survey performed in Europe, Asia, Australasia and North America showed that although 45 % of ICUs practice antibiotic nebulization, very few actually follow the recommendations [28]. Given the commonness of use of aerosolization in critical care, yet the uncertainty over the optimal approach for administration, this article aims to discuss the essential concepts related to aerosolized drug therapy in critical care.

### The aerosol system

An aerosol is defined as a suspension of liquid or solid in a gaseous medium [29]. For successful aerosolization, consideration of the aerosol system is required. The aerosol system includes the drug, the aerosol device, the disease (i.e. the target site) and the patient’s respiratory system, with the ventilator being an additional factor in mechanically ventilated patients. The aim of the aerosol system is to produce aerosols with characteristics suitable for drug delivery to the lungs. Drug deposition, absorption, metabolism and elimination are essential determinants of the pharmacokinetic profile resulting from the aerosol system.

Key expressions used to evaluate the aerosol system performance include [30] the following:

- Emitted dose (ED)—the amount of drug exiting the delivery device.
- Fine particle fraction (FPF)—the mass of particles below a cut-off diameter [31].

The overall efficiency of the aerosol system is a composite of the ED, the dose delivered to the lung (FPF as a surrogate marker) and lung bioavailability. The ED and FPF are normally determined in vitro and are governed by particulate properties and device design. The bioavailability of the drug is influenced by patient factors such as airway and lung anatomy, drug permeability across membranes, metabolism of the drug and phagocytic clearance in the lung [32] as well as FPF.

### Aerosol deposition

**Fig. 1:**
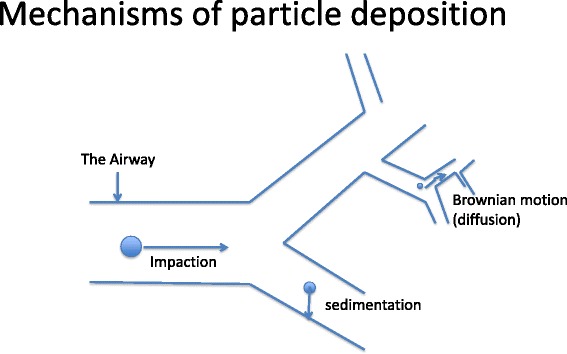
Mechanisms of particle deposition

The efficacy of the aerosolized drug depends on the dose deposited at the target site of action as well as its distribution in the lungs [33]. Deposition in the airways can occur by inertial impaction, gravitational sedimentation or diffusion (Brownian motion) (Fig. 1). Because of the turbulence and high air velocity associated with aerosolization, an inertial impaction method is predominant in the first 10 branchings of the airway [34]. This proximal region is the target for aerosol therapy for diseases such as COPD, asthma and ventilator-associated tracheobronchitis. In the distal five to six airway generations, however, sedimentation predominates due to lower air velocity [34]. At the alveolar level, minimal air velocity means no effect of impaction will occur and a combination of sedimentation and diffusion will influence drug deposition [34]. Most aerosolized particles for therapeutic purposes are in the range of 2–5 μm and diffusion is the predominant mechanism for lung deposition. The optimal technique for aerosolization is important to achieve distal airway and alveolar deposition.

## Factors affecting aerosolized drug delivery in the critically ill patient

**Fig. 2:**
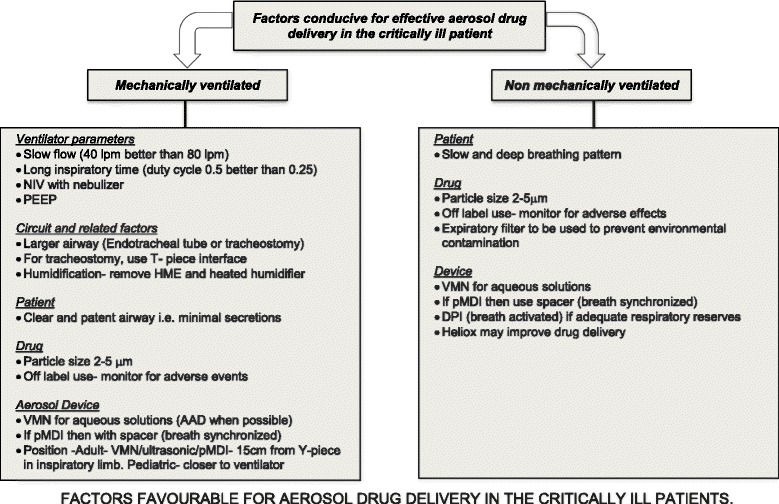
Factors favourable for aerosol drug delivery in critically ill patients. Figure derived from references [19, 20, 25, 29, 31, 38, 45, 51, 81, 82, 91, 93, 130]. *NIV* non-invasive ventilation, *HME* heat and moisture exchanger, *pMDI* pressurized metered dose inhaler, *AAD* adaptive aerosol device, *VMN* vibrating mesh nebulizer, *DPI* dry powder inhaler, *PEEP* positive end-expiratory pressure

Drug concentrations in lung tissue are affected by the aerosolized dose administered, patient factors, device factors and the formulation of the drug. Mechanical ventilation (MV) introduces additional elements such as the circuit and the ventilator and associated factors. For the purposes of describing the factors affecting aerosol therapy, critically ill patients could be classified into two groups: ventilated patients and non-ventilated patients [35–38]. Figure 2 shows the factors conducive for effective aerosol drug delivery in the critically ill mechanically ventilated and non-mechanically ventilated patient groups.

### General factors affecting aerosolized drug delivery

#### Airway anatomy and physiology

Airflow and tidal volume influence the effect of airway anatomy on aerosol deposition. Patients suffering from airway obstruction such as asthma or COPD have impaired mucociliary clearances and mucous retention [39]. For drugs with poor trans-mucous permeability (e.g. aerosolized aminoglycosides) this could mean reduced drug delivery and hence impaired efficacy, although this is yet to be confirmed in clinical studies [40].

Chronic inflammation may result in airway remodelling, which changes the dynamics of airflow [33, 35], and impaired mucociliary clearance, thus reducing the pulmonary drug deposition [33, 41]. These changes lead to a proximal shift in the airway deposition pattern of the aerosols [42].

*Significance—*Abnormal airways and impaired mucociliary clearance serve as a barrier to effective aerosolized drug therapy when the target site is the lung parenchyma.

#### Regional lung aeration

The airflow is not homogeneous throughout the lungs even in health. The result in an upright patient is that the apical portions of the lungs receive lung deposition of the order of a 2:1 higher ratio compared with the basal regions [43]. This difference is significantly reduced in the supine position [44]. Moreover, most lung diseases are regional which adds to the heterogeneity to regional airflow, an important determinant of aerosol deposition [45]. For example, it has been shown that there is lower deposition in areas of poor air flow (i.e. atelectatic lungs) [46].

**Fig. 3:**
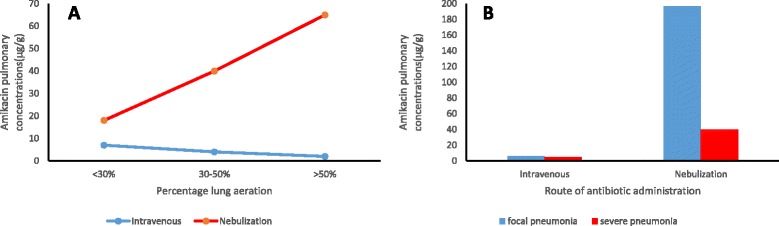
Effects of regional lung aeration and pneumonia on drug concentration in lungs. **a** Relationship of lung aeration (%) to pulmonary concentration of amikacin (μg/g) for different routes of administration. **b** Relationship of route of drug administration to pulmonary concentration of amikacin (μg/g) for different severities of pneumonia. Pulmonary concentrations derived from homogenized lung tissue specimens measured by an immunoenzymatic method. Figure derived from Elman et al. [47]

In the area of antibiotics, there is a large body of work with experimental pneumonia models which have demonstrated that lung tissue concentrations of nebulized amikacin, using a ultrasonic nebulizer, was significantly higher than the concentrations resulting from administration via the intravenous route [47, 48]. Indeed, even though deposition of nebulized drug decreased with more severe pneumonia, it still resulted in higher lung tissue concentration than that achieved from intravenous administration. Figure 3 illustrates this phenomenon. The same group also demonstrated that nebulized amikacin resulted in greater bactericidal activity leading to greater sterility rates compared with the intravenous route [49]. When compared with continuous intravenous infusion of ceftazidime, frequent nebulization achieved higher lung tissue concentrations with better bactericidal effects in an experimental model of *Pseudomonas* pneumonia [50].

*Significance*—Differences in regional lung aeration may explain some of the variability in therapeutic outcomes amongst different lung diseases.

#### Inhalation patterns

In a critically ill, spontaneously breathing patient, air flow is likely to be turbulent leading to impaction in the proximal airway. For drugs dependent on lung deposition for their effect, this results in a decreased pharmacological effect. In contrast, laminar flow patterns are considered to enable optimal lung deposition [51]. In the critically ill patient, certain MV settings (e.g. square wave airflow pattern) enable generation of laminar airflow to improve drug deposition in the lungs.

On the other hand, lower flows may reduce the ED when dry powder inhalers (DPIs) are used [52]. Using pressurized metered dose inhalers (pMDIs) with valved holding chambers (VHCs) or spacers could mitigate this effect.

*Significance*—Whilst a laminar flow pattern would be beneficial for aerosolized drug delivery, mechanistic data need to be confirmed using clinical trials.

#### Surfactant

Diseases such as pneumonia and other inflammatory lung diseases result in deficiencies of lung surfactant both in content and/or effect [53, 54]. Drugs with high solubility will probably have a uniform dispersion compared with insoluble drugs. Inferentially the soluble drugs are likely to have longer and more effective lung residence times, thus improving drug potency [55]. Surfactant deficiency is associated with atelectasis, which in turn impairs drug deposition [42]. Studies on surfactant replacement therapy in acute lung injury and ARDS, however, have failed to demonstrate benefit and may even be deemed harmful [56, 57]. Aerosolized surfactant therapy has been studied as a mucokinetic agent in specific conditions [58]. Its application for this purpose in critically ill patients needs further study.

*Significance*—Uncertain benefits requiring further studies to demonstrate effects of surfactant.

#### Mucous barrier and atelectasis

A major fraction of the aerosolized drug is entrapped in the mucous in the conducting airways. Factors such as particle size, solubility, lipophilicity and charge govern the ability of the drug to penetrate this mucous barrier. For example, steroids and antimicrobial agents are seen to have reduced trans-mucous transport [39, 40]. Atelectasis is a common occurrence in the majority of critically ill patients. This may have adverse effects on drug deposition and may result in heterogeneous distribution in the lung [59].

*Significance*—Both, mucous and atelectasis serve as a barrier to effective aerosolized drug therapy.

#### Device effects

A detailed discussion on the effect of device-related factors has been reviewed elsewhere [20, 60]. Appropriate particle sizes are important to enable adequate concentrations at the target site. Particle size also determines the mechanism of deposition in the respiratory system [31]. Particles that distribute deep in the smaller airways (<5 μm) are reported to have up to 70 % deposition efficiency [33, 61]. Smaller particles (1–3 μm) are considered to have the optimal droplet size for efficient deposition in the alveolar airspaces, for systemic delivery [62]. In this regard, the efficiency of the aerosol device can be defined to be the ability to generate the aerosol in the desired particle size range.

pMDIs with spacers or VHCs have demonstrated superior deposition efficacy over nebulizers in various studies [63–65], although the VHCs cannot be used for mechanical ventilators due to their inability to trigger/activate the device. DPIs have no propellant, are inherently breath-synchronized/activated and produce little variation in particle size. These features may make DPIs the preferred delivery device. In critically ill patients, however, poor respiratory reserve and diminished patient efforts are barriers to achieving the desired respiratory pattern for effective use of DPIs. The DPIs also vary widely in their efficacy [66] and their use in mechanically ventilated patients is not typically possible with a standard set up [67]. Thus, DPIs are presently used in stable and unventilated patient groups. Both pMDIs and DPIs are limited by the formulations available to be delivered by these devices.

Nebulizers are different devices that are used to transform liquid formulations and suspensions into an aerosol form. These devices can be used to deliver larger volumes of a drug as an aerosol either intermittently or continuously, for prophylaxis or treatment purposes. Depending on their mechanism of operation, there are three types of nebulizers: jet, ultrasonic and SMNs. Jet nebulizers are the cheapest and simplest, albeit being inefficient in drug delivery [68]. Their drawbacks are noise, poor dosing control and the requirement for changes in the ventilator settings such as airflow and tidal volume; although improvements have been made in the form of reservoirs and new baffles that ensure more optimal particle sizes. Breath-enhanced versions of the jet nebulizers could increase FPF, improve drug delivery and reduce drug loss. There are limited studies evaluating the efficiency of these newer jet nebulizers and data are certainly lacking in critical care settings [69]. Newer ventilators have in-built nebulization systems which improve the efficiency by synchronizing nebulization with the respiratory cycle. Ultrasonic nebulizers are infrequently used and also have limitations [19, 70]. They are expensive, large in size, increase concentration of the drug during nebulization and can cause thermal inactivation of the nebulized drug. Mesh nebulizers are the result of improvement in nebulizer technologies. Although more efficient and with significant advantages, there is a dearth of human studies using mesh nebulizers. Despite major improvements in the technology there is a need to reduce the cost of these devices. Table 1 compares and contrasts the principles, advantages and disadvantages of different nebulizers.

**Table 1:** Comparison of different types of nebulizers

Nebulizer type	Mechanism of action	Types	Advantages	Disadvantages
Jet [68]	Pressurized gas forms a jet passing through a tube creating a low-pressure zone (Venturi effect) that draws liquid formulation into the jet stream (Bernoulli effect)	• With a corrugated tube	• Cheap	• Inefficient
		• With a collection bag	• Easy to use	• Difficult to clean
	Droplet size > 5 μm	• Breath-enhanced jet nebulizers	• Effective in delivering drugs that cannot be delivered with pMDIs and DPIs	• Need compressed gas and additional tubing
		• Breath-actuated jet nebulizers	• Breath-enhanced and breath-actuated options	
Ultrasonic [70, 131]	Piezoelectric crystal converts an electrical signal into high-frequency vibrations in the liquid, forming an aerosol using cavitation and capillary mechanisms	• Small volume (e.g. for medications)	• Easy to use	• Large residual volume
		• Large volume (e.g. for hypertonic saline used for sputum induction)	• More efficient than jet nebulizers	• Unable to nebulize viscous solutions
	Drug output alpha vibration amplitude		• Shorter nebulization time (better for large volumes)	• Degradation of heat-sensitive materials—so inappropriate for proteins
	Particle size alpha vibration frequency			
	Droplet size variable, may be less than 5 μm			• Aerosol temperature 10–14 °C higher than that in jet nebulizer
				• Large device size
Vibrating mesh [19, 70]	Aerosol is produced by forcing the liquid using the micropumping action through the vibrating mesh containing funnel-shaped holes	• Active (e.g. Aeroneb®; Aerogen, Galway, Ireland)	• Silent operation, portable	• More expensive
	Droplet size < 5 μm			
		• Passive (e.g. Microair NE-U22®; Omron, Bannockburn, IL, USA)	• Short treatment time	• Cleaning can be difficult
			• Minimal residual volume	• Drug dose needs to be adjusted in transition from jet nebulizers
			• Self-contained power source	• Inability to use to aerosolize viscous liquids
			• Optimize particle size for specific drugs	• Inability to aerosolize drugs that crystallize on drying
			• More output efficiency than other nebulizers	
			• Two to three times higher drug deposition compared with jet nebulizers	
			• Aerosol temperature usually unchanged	
			• Unchanged osmolality	
			• Easy to use	

*pMDI* pressurized metered dose inhaler, *DPI* dry powder inhaler

*Significance*—Where possible, pMDIs with spacers should be used. DPI use is likely to be limited in critical care. For nebulizers, the device should be selected according to the formulation used and the desired site of deposition and effect.

#### Drug effect

The rate and extent of absorption of the aerosolized substances is dependent on the molecular weight, pH, electrical charge, solubility and stability.

Macromolecules < 40 kDa are observed to be better absorbed (in minutes) in the bloodstream following inhalation in the airways (e.g. insulin, molecular weight (MW) 5.7 kDa) [71]. However, macromolecules > 40 kDa are absorbed slowly over hours (e.g. albumin, MW 68 kDa) [72]. Molecules with MW > 30 kDa may need an absorption enhancer for absorption in the alveoli [73].

*Significance*—Depending on the desired site of action, appropriate drug formulations should be used alongside delivery devices that would generate a suitable particle size.

#### The Heliox effect

A combination of helium and oxygen (Heliox) reduces gas density and increases aerosol deposition, particularly in the peripheral lung [74]. With pMDIs, Heliox has been reported to increase aerosolized drug delivery during MV [75]. However, with jet nebulizers Heliox also increases the nebulization time, requiring higher gas flows to compensate for the low-density gas [76]. In an experimental study, there was no increase in lung deposition of nebulized ceftazidime in bronchopneumonic lungs compared with healthy lungs [77].

*Significance*—Further investigations and large-scale trials are needed to evaluate the effect of Heliox in critical illness.

#### Sputum antagonism

Because of a variety of proposed mechanisms, patient sputum is thought to cause aminoglycoside inactivation resulting in ‘sputum antagonism’ [78].

*Significance*—Uncertain, therefore the effect of sputum antagonism requires further in-vivo investigation. Current data from CF patients support use of inhaled aminoglycosides [79, 80].

### Specific factors affecting aerosolized drug delivery in mechanically ventilated patients

Aerosol therapy is routinely used in mechanically ventilated patients, both invasive and non-invasive, and is inherently challenging due to the interplay of a variety of factors [25]. However, not all nebulization techniques are comparable. The patient position, formulation, temperature, endotracheal tube size, presence of airway obstruction or ventilatory asynchrony, flow pattern, respiratory rate, dose and frequency applied or position of the nebulizer in the circuit are important factors that influence delivery to the lung. The higher the turbulence, the lower the drug deposition in the distal airways. Optimal settings of nebulization are not tolerated by many patients (such as those with severe hypoxemia, associated with ARDS or pneumonia) and require the addition of deep sedation and relaxation, which prolongs the duration of MV. Disposition in unilateral pneumonia might be imbalanced.

#### Type of aerosol generator in the circuit

Currently, nebulizers and pMDIs, with and without spacers, are two types of devices available for use in mechanically ventilated patients. Depending on the site of action, devices producing an appropriate particle size should be used [81].

Nebulizers take a considerably longer time to deliver a standard dose as compared with other devices. There is also a variation in efficiency between nebulizer types and between nebulizers in different batches [20]. This effect is accentuated when coupled with the effects of different ventilator modes and lung mechanics [82]. Inadequate cleaning and disinfection of the nebulizer increases the risk of nosocomial pneumonia [83]. Compared with jet nebulizers, VMNs could increase the drug delivery by 2–4-fold [19], although as discussed previously the nebulizer choice is dependent on the formulation and the desired delivery site.

pMDIs are easy to administer, require less staff time, provide reliable dosing and have minimal risk of bacterial contamination when compared with nebulizers. When used with a collapsible spacer in the circuit, the circuit does not need to be disconnected [25]. pMDIs are also more cost-effective than nebulizers [84]. Although only bronchodilators and anti-inflammatory agents are available for this device, it is seen that using pMDIs significantly reduces overall costs of care and could be equally effective in the treatment of inflammatory airways disease such as asthma and COPD [20, 84–88]. In-vitro studies have shown improved aerosol delivery with large spacers compared with that with small spacers for pMDIs and VMNs [89]. Published recommendations for the correct methods of their use are available [25]. Others have shown modest improvement in the aerosol delivery [90].

*Significance*—pMDIs are possibly more effective than nebulizers. VMNs appear superior to other nebulizer types although the choice should be dependent on the drug formulation properties and the desired deposition site. At this time, there is insufficient evidence to support the use of either delivery method over the other [91]. The use of spacers with pMDIs needs further clinical trial to test the efficacy.

#### Position of the aerosol generator

In-vitro studies [92] using adult ventilators have shown that, when using vibrating mesh and ultrasonic nebulizers as well as the pMDI, a position 15 cm from the Y-piece in the inspiratory limb of the circuit yields the highest drug delivery. In a constant flow pattern of ventilation, the VMN connected to the endotracheal tube could be as effective [93]. However, jet nebulizers seem to perform better when positioned closer to the ventilator, possibly due to the effect of the continuous gas flow ‘charging’ the circuit, which functions as an aerosol reservoir [92]. For non-invasive ventilation (NIV), using the VMNs position after the exhalation port is more efficient for drug delivery compared with that before the exhalation port [94].

*Significance*—The best position for the aerosol generator may be 15 cm from the Y-piece in the inspiratory limb. In-vivo studies are required to make definitive conclusions.

#### Effect of tracheostomy and airway size

Although the endotracheal tubes and tracheostomy tubes present certain similarities, the tracheostomy tube is shorter and more curved than an endotracheal tube. In patients who are not mechanically ventilated, a T-piece interface between the tracheostomy tube and the nebulizer has been demonstrated to be more effective than a tracheostomy mask [95, 96].Preferably, the inner cannula should be removed before nebulization particularly for the smaller sized tubes [97] because smaller diameter airways lead to an increase in the resistance to airflow, resulting in increased drug deposition in the artificial airways and tracheobronchial region [98, 99].

*Significance*—For aerosolized drug delivery, larger size artificial airways are better.

#### Heat and humidity of the circuit

In mechanically ventilated patients, a temperature of 34–41 °C (average 37 °C) and relative humidity of 95–100 % are required to prevent heat loss [100]. Humidification also prevents drying of secretions, mucous plugging and consequently atelectasis. There are two major methods of humidification—active and passive. Active methods include a heated humidifier (HH) and passive methods include a heat and moisture exchanger (HME).

Humidification is thought to have a significant effect on aerosol drug delivery. Because of the hygroscopic effects of humidification, there may be a 2–3-fold growth in particle size as they pass through airways. This increase in size may reduce peripheral lung drug deposition and hence pharmacological efficacy [101]. Compared with humidified conditions, drug delivery can be doubled in non-humidified conditions [92]. It is recommended that HH should be ceased for the duration of therapy. Of interest, in an in-vitro non-mechanically ventilated model, using the excipient enhanced growth (EEG) of sub-micrometre particles, one group has demonstrated increased aerosol deposition in the airways and lungs [102, 103]. Further investigations of this method are required to harness its effect in MV.

The HME is a physical barrier and should not be placed between the delivery device and the patient. The particulate air filter in the expiratory limb, used to protect the ventilator and the flow meter, could get saturated resulting in airflow obstruction. It is recommended that the filter should be changed after every nebulization treatment [18, 26, 28].

*Significance*—Using HME or a particulate air filter with nebulization could result in air flow obstruction. Awareness and routine changing of air filters after each nebulization should be performed.

#### Breath characteristics

Ventilator breath characteristics have an important effect on the efficacy of aerosol delivery. Slower inspiratory flows, long inspiratory times [104] and tidal volumes > 500 ml (using a pMDI) [105] correlate well with improved aerosol delivery. Higher bias flow is seen to reduce the delivery efficacy of nebulizers [19]. Decelerating flow pattern is considered inferior to constant flow pattern for drug delivery [93]. The effect of ventilation mode is negligible for pMDIs [16]. The delivery efficiency in patients on NIV is seen to be comparatively less [106]. However, it must be remembered that specific techniques of ventilation may in themselves produce a greater benefit than the relative detriment of drug delivery (e.g. in NIV and asthma). Hence, in acute asthma, NIV plus nebulization is more effective than nebulization alone [107].

A prescribed ventilatory pattern may not be practical in the critically ill patient. The most effective combination of tidal volume, flow and other ventilator parameters for aerosol delivery can be calibrated to the drug and delivery device using in-vitro models [108].

*Significance*—Tidal volumes > 500 ml may enhance aerosolized drug delivery. NIV results in effective therapy despite reduced drug delivery in conditions like asthma. Ventilator settings optimal for nebulization, however, could lead to patient–ventilator dyssynchrony in severely hypoxemic patients (e.g. due to severe pneumonia)—thus requiring deep sedation, which may increase the duration of MV.

#### Effect of positive end-expiratory pressure

Positive end-expiratory pressure (PEEP) is a commonly used ventilator setting as part of the lung protective ventilatory strategy in severe lung diseases [109]. PEEP has significant effects on regional ventilation and perfusion [110] and hence could influence the PK of an aerosolized drug. In an animal model using radiotracers, PEEP was found to enhance aerosol clearance. This could be due to the stretching of the alveolar epithelium and enhancing the distribution of aerosol into the bloodstream [111].

*Significance*—PEEP is potentially beneficial, although further data are needed to quantify the effect on aerosolized drug delivery.

#### Effect of drugs

The choice of one antimicrobial against another should consider efficacy data, costs, local antimicrobial resistance patterns and drug availability. Aminoglycosides require tissue concentrations >10-fold higher than the MIC to be maximally effective. Because airway inflammation could increase systemic absorption and the molecular weight is low, serum aminoglycoside concentrations should be monitored to avoid systemic toxicity. Beta-lactams are rapidly cleared from airways, requiring frequent administration. Colistin is administered in its anionic (methanesulfonated) form—colistimethate. Despite high doses (up to 1 million units of colistimethate every 8 hours (80 mg of colistimethate, equivalent to 33 mg of colistin base)) as administered in colonized patients with bronchiectasis, lung epithelial lining fluid concentrations are not above 4 mg/L after 8 hours (upper threshold of EUCAST MIC breakpoint for *Pseudomonas*) or even above 2 mg/L after 8 hours in many patients (EUCAST MIC breakpoint for *Klebsiella* sp*.* and *Acinetobacter baumannii*)*.* Therefore, high doses (5 million units every 8 hours) should be considered in pneumonia.

#### Effect of dosing

Despite delivery of drugs via the inhaled route, significant extrapulmonary drug losses may mean that the actual amount of drug delivered might be less than 60 % of the ED into the trachea and even less will reach the alveolar space [112]. This factor should be taken into account when calculating dosing regimens. A number of animal studies have been useful to better understand the mechanistic principles of aerosol therapy. Guillon et al. [113] showed effective teicoplanin nebulization during MV with good PK properties compared with the intravenous route. Others successfully nebulized ceftazidime to achieve high local concentrations [77, 114].

Further studies are required to quantify the exact dosing amount and schedule using PK studies. Doses should be different in patients with colonization, tracheobronchitis or pneumonia. Increasing doses (e.g. 5 million units of colistin) require longer periods of nebulization (~1 hour) which is not well tolerated by patients suffering from ARDS.

*Significance*—The inhaled drug dose is likely to be significantly higher than expected due to concerns about drug losses. Further PK–PD studies are required to guide inhaled drug dosing.

#### Effect of timing of nebulization

Most of the drug losses occur in the exhalation phase of ventilation. To minimize this loss, the actuation of the inhaler or nebulizer could be matched with inspiration [17]. However, the use of the spacer–pMDI combination negates the effect of lack of breath synchronization [105].

The effect of breath synchronization on aerosol deposition is unproven. Using radiolabelled aerosols, Dubus et al. [115] showed that there is no significant increase in aerosol deposition in neonatal ventilation with breath synchronization. Further investigations are thus needed to evaluate the effects of breath synchronization on aerosol deposition. In any event, devices which introduce synchronization of drug delivery facilitate tolerance.

*Significance*—Breath actuation of the drug delivery devices has the potential to improve drug delivery. However, trial-based data are required to establish efficacy in aerosolized drug deposition.

### Effect of high-flow nasal cannula

High-flow nasal oxygen therapy is becoming a widely prevalent therapy in intensive care [116]. A number of factors influence the nebulization therapy in patients using high flow, which was studied recently in an in-vitro model [117]:

1. Position of the nebulizer—a position distant from the humidifier (closer to the patient) improved delivery of the drug upstream.
2. Nebulizer type—VMNs demonstrated improved delivery as compared with jet nebulizers, although the nebulizer choice is dependent on the formulation and desired site of action.
3. Airflow—the delivery of respirable mass is lower with higher airflow and improves at a lower airflow.
4. Patient efforts—converse to the effect of airflow with a high-flow oxygen system, in situations mimicking respiratory distress (i.e. increased patient inspiratory airflow) the delivery was in fact better. An open mouth, on the contrary, had no significant difference to closed mouth with respect to drug delivery.

*Significance*—Limited data suggest better drug delivery using VMNs at a lower airflow even in patients with respiratory distress. Further in-vivo studies need to be performed using high-flow oxygen therapy devices.

### Contemporary applications of aerosol therapy in critical care: focus on antibiotics

Table 2 summarizes the common applications of aerosol therapy in critical care.

**Table 2:** Common applications of aerosol therapy in intensive care^a^

	Drug class				
Feature	Bronchodilators	Anti-inflammatory	Antimicrobial agents	Vasoactive agents	Heliox
Indications	Bronchospasm (e.g. acute asthma, COPD exacerbation)	Airway inflammation (e.g. acute asthma or COPD exacerbation, acute interstitial lung disease)	MDR tracheobronchitis MDR pneumonia	Right ventricular failure	Asthma
			*Aspergillus* prevention (lung transplant)	Pulmonary hypertension	
Site of action	Airways	Airways or alveoli	Airways or alveoli	Alveoli	Airways
Preferred device	pMDI with spacer	pMDI with spacer	VMN	VMN	
Drugs	Beta-agonists (e.g. salbutamol, salmeterol)	Budesonide	Antibiotics (e.g. tobramycin, colistin, amikacin, ceftazidime, amphotericin B)	Epoprostenol	Helium
	Anticholinergics (e.g. ipratropium, tiotropium)	Fluticasone		Iloprost	
Formulations available	Yes	Yes	Some	Yes	

Table derived from references [19, 24, 37, 87, 132–151]

^a^Anaesthetic gases were not in the objectives of this analysis

*Heliox* helium and oxygen, *COPD* chronic obstructive pulmonary disease, *MDR* multidrug resistant, *pMDI* pressurized metered dose inhaler, *VMN* vibrating mesh nebulizer

Aerosolized bronchodilators and corticosteroids have been effectively utilized in critical care. Aerosolized antibiotics are quickly gaining more data to support their position in the critical care armamentarium. With improvements in drug formulation and delivery devices, more is now known about the optimal conditions required for effective aerosolized therapy as summarized in Fig. 2.

**Fig. 4:**
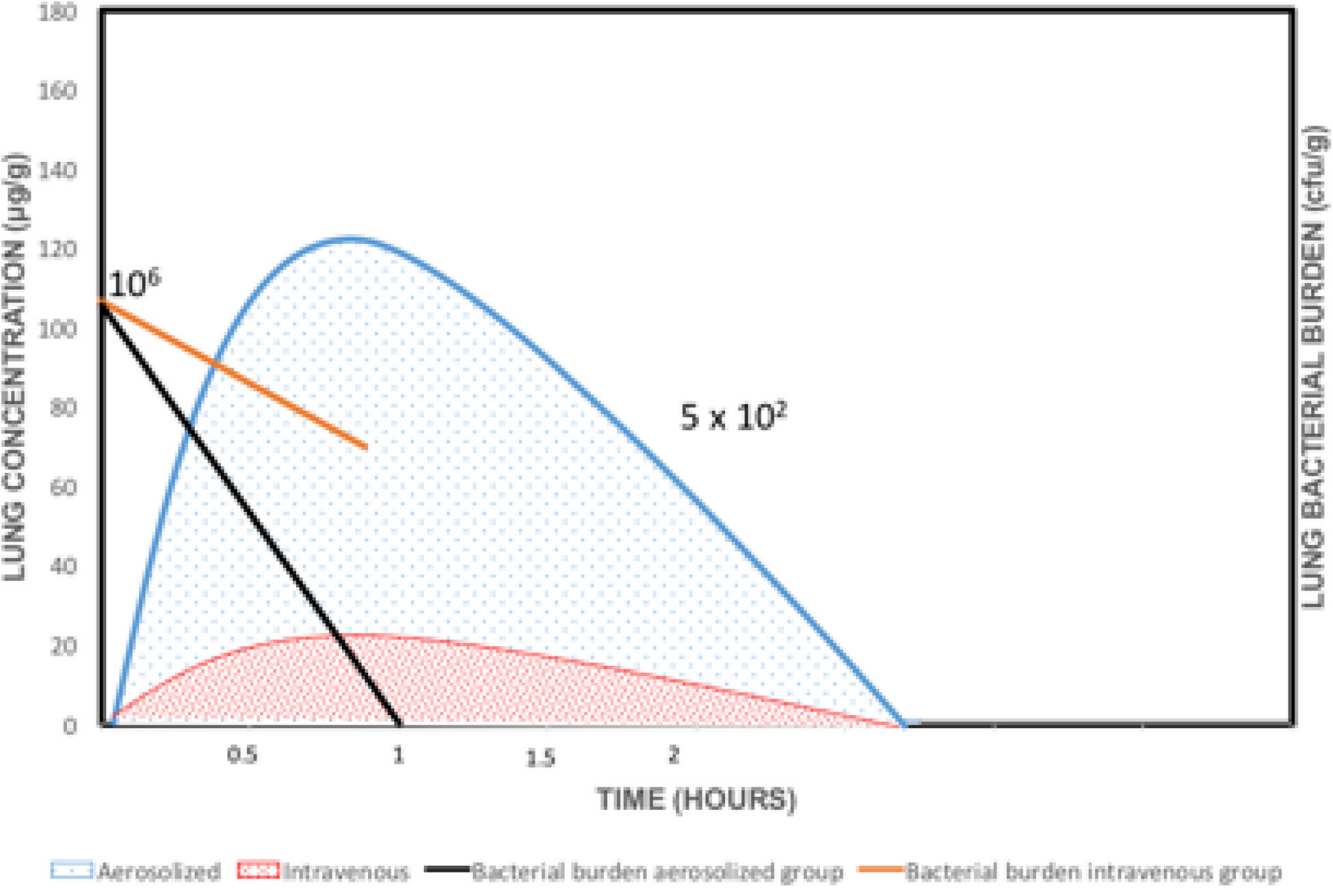
Comparison of lung concentration (measured by HPLC) of amikacin between aerosolized and intravenous administration. Measurement done 1 hour after the second administration performed 48 hours after bacterial inoculation. Diagram derived from the data of Goldstein et al. [49]

Despite these developments there are concerns that best evidence for administration is not being applied, particularly for aerosolized antibiotic therapy [118, 119]. Clinical and experimental study data for aminoglycosides and colistin are perhaps most numerous for antibiotics in critical care [28]. Aminoglycosides are concentration-dependent antibiotics whereby the bactericidal effect is best described by the C_max_/MIC ratio. Studies have shown that intravenous aminoglycosides penetrate poorly into the epithelial lining fluid [48, 120]. In an *Escherichia coli* inoculation pneumonia model, aerosolized amikacin was seen to achieve significant lung concentrations [48]. Figure 4 is an illustration of this phenomenon. On the other hand, with repeated administration, there was no accumulation effect and hence no toxicity concerns with aerosolized amikacin [46]. In experimental studies, the serum concentration of amikacin was higher when aerosolized amikacin was used in a pneumonia model [48] compared with that of healthy lungs [46]. Moreover, a combination of intravenous and aerosolized aminoglycosides has not been shown to increase cure rates compared with that of aerosolized antibiotic alone. Thus, for the treatment of ventilator-associated pneumonia, aerosol therapy alone may be adequate without the need for intravenous therapy, decreasing the risk of systemic toxicity [121].

**Fig. 5:**
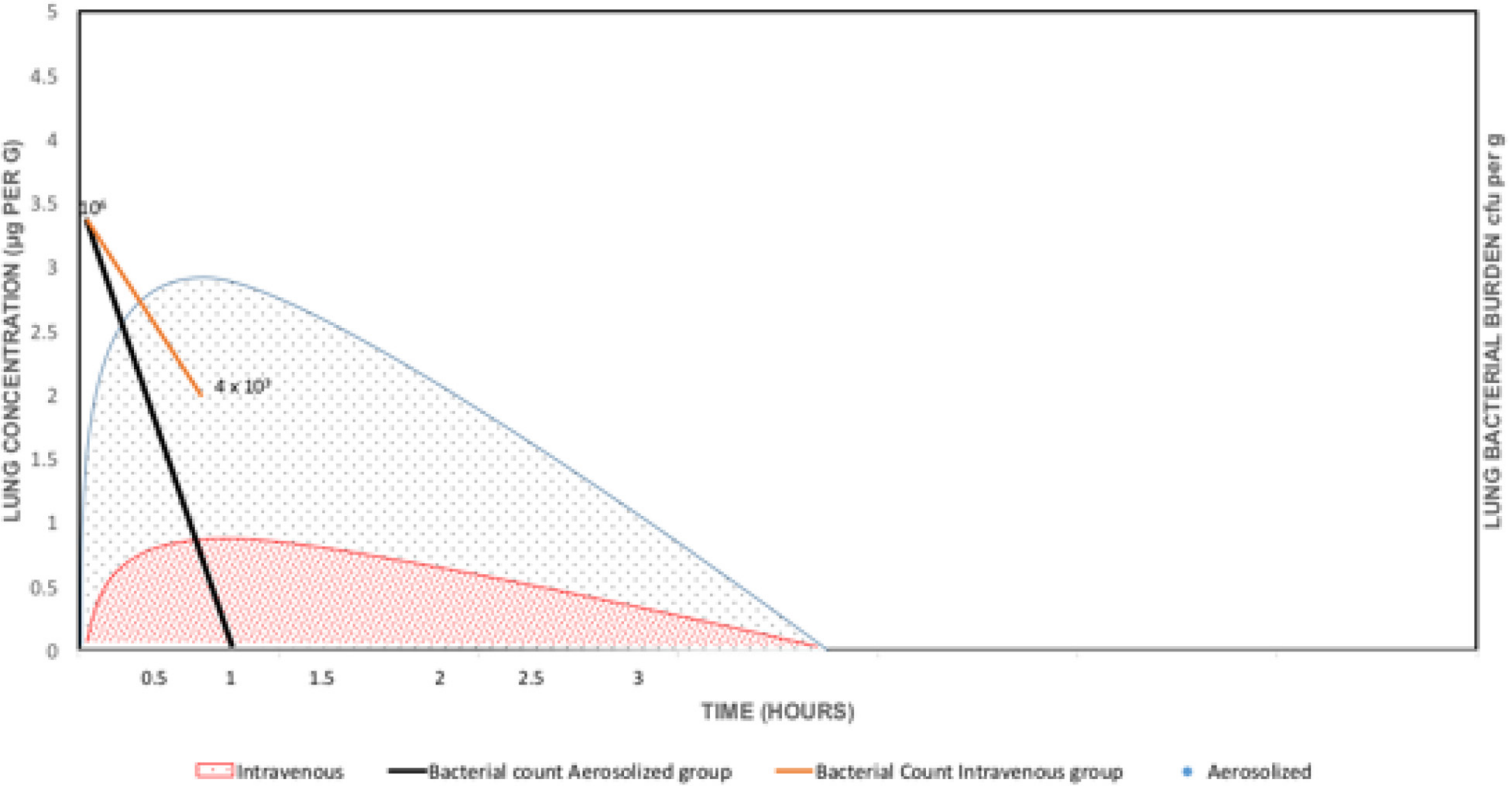
Comparison of lung concentration (measured by HPLC) and bacterial burden of colistin between aerosolized and intravenous administration. Samples taken 1 hour after the third aerosol in the aerosol group and the fourth infusion in the intravenous group and 49 hours after the bacterial inoculation. Diagram derived from data of Lu et al. [112]

Colistin, also a concentration-dependent antibiotic, is another antibiotic used widely in aerosolized form. Colistin aerosolization is not approved by the FDA and is not licensed for human use in China. Like aminoglycosides, colistin has poor lung penetration when given intravenously. Experimental studies have shown that a rapid and high bactericidal effect can be achieved with aerosolized colistin [112]. Figure 5 illustrates this phenomenon. As demonstrated by Lu et al. [112], with low serum concentrations resulting from aerosolized colistin in an inoculation pneumonia model, the risk of toxicity is minimal. In a prospective observational study, Lu et al. [121] demonstrated similar clinical cure for patients with VAP where susceptible *P. aeruginosa* or *A. baumannii* were treated with only intravenous colistin and MDR strains were treated with nebulized colistin. Combined intravenous aminoglycoside and aerosolized colistin has not been shown to be superior to aerosolized colistin alone although implemented worldwide. The benefit from the use of aerosolized colistin instead of systemic colistin is to avoid nephrotoxicity, and this was further confirmed in one randomized clinical trial [122].

### Limitations of aerosol therapy in intensive care

Observational cohort studies report less adverse events than randomized clinical trials. Indeed, there is potential to cause systemic toxicity (e.g. nephrotoxicity by aminoglycosides) or local toxicity in the form of airway irritation, cough and often bronchospasm [123], worsening hypoxemia (and secondary arrhythmias) as well as pulmonary injury when using aerosol therapies [124]. Ventilator malfunction and obstruction of expiratory filters have been reported and contraindicate the use of drugs with lipid components or lactose sugar in the formulation (such as zanamivir or lipid-based amphotericin formulations), and careful monitoring of the potential increase of airway pressure and oxygen saturation is required to anticipate severe adverse events [125]. Modification of ventilator parameters for appropriate jet nebulizer use (Table 3) is not tolerated by some patients, increasing the work of breathing and ventilator dyssynchrony (requiring additional sedation). Poor tolerance may preclude the use of aerosolized antibiotics in patients with PaO_2_/FiO_2_ < 200 mmHg or high PEEP requirements.

**Table 3:** Optimization of ventilator parameters required for aerosolization of antibiotics modified from Lu et al. [121]

• Nebulizer placement—in the inspiratory limb 10 cm proximal to Y-piece	
• Diluted in 10 ml saline	
• Remove HME filter	
• Ventilation mode—volume controlled	
• Airflow pattern—constant inspiratory flow	
• Ventilator settings—RR 12/minute, 50 % I: E ratio, VT 8 ml/kg	
• End-inspiratory pause, 20 % duty cycle	
• Delivered over 60 minutes	
• Expired aerosolized particles collected in a filter	

*HME* heat and moisture exchanger, *RR* respiratory rate, *I:E* inspiratory: expiratory ratio, *VT* tidal volume

Initial concerns regarding drug resistance as a result of intratracheal or nebulized use of antibiotics (polymyxin B) have been investigated and do not appear to be supported, with aerosolized antibiotics using newer devices no more likely than intravenous therapy to confer bacterial resistance [126]. This was probably linked to previous-generation nebulizers and the technique of administration (instillation, pharyngeal aerosolization, etc.). However, this finding must be interpreted reservedly because no long-term follow-up has been performed.

Tolerance of aerosolization is different when drugs are nebulized for different durations of time. As a consequence, this might limit use of aerosolization in patients with ARDS or severe hypoxemia, such as severe pneumonia (in contrast to ventilator-associated tracheobronchitis), who often have poor tolerance. When high doses of colistin are nebulized, the infusion volume may represent an hour of nebulization and many patients require added sedatives or relaxation (with potential increased risk of myopathy or hypotension). This requirement would be associated with a prolonged MV period and extra length of stay [125]. Further clinical trials should therefore use pre-defined outcome parameters (rather than surrogates), control by hypoxemia and careful recording of adverse events.

Environmental contaminations resulting from aerosolization of drugs in an open circuit system pose a small but significant risk to the caregivers. Using expiratory filters with valves in the aerosol delivery devices could minimize this. This occupational risk exposure should be assessed and interventions to mitigate the risks should be implemented [127]. When using aerosolized antibiotics, it is recommended to change the filter after every therapy.

Optimizing the aforementioned factors could lead to effective drug delivery. However, it is important to realize that aerosolization of medications does not automatically lead to beneficial drug effects and may in fact be harmful, as shown in some studies [128, 129].

## Conclusions

Aerosol therapy provides effective drug delivery in the critically ill patient. Careful consideration of the various elements that affect pharmacological effect of aerosolized therapies is essential to derive optimal therapeutic benefit. Effective drug delivery alone does not ensure successful aerosol drug therapy. It is crucial that the drug in its aerosolized form should have efficacy in the specific disease condition to derive clinical benefit.

Good quality data and clinical experience support use of bronchodilators such as salbutamol, anti-infectives such as tobramycin, aztreonam and colistin, and anti-inflammatory agents such as budesonide. Although with application of principles it is possible to provide aerosol drug delivery, the effectiveness of the therapy in disease conditions is yet to be proven. This is because there is a scarcity of high-quality trial-based data in this area to quantify how effective these agents are in the critically ill patient.

Given the challenges of effective treatment of the critically ill patient, it is necessary to optimize as many factors as possible for effective drug delivery. Hence, it is important that guidelines for aerosol therapy are developed. It is envisaged that as the technologies become mature through rigorous evaluation, a diverse range of aerosol therapies with unique advantages (i.e. controlled release/sustained release or direct targeting) and or for specific indications may be possible.

## Abbreviations

ARDS: Acute respiratory distress syndrome
CF: Cystic fibrosis
COPD: Chronic obstructive pulmonary disease
DPI: Dry powder inhaler
ED: Emitted dose
EUCAST: European Committee on Antimicrobial Suceptibility Testing
FDA: Food and Drugs Administration
FiO2: Fraction of inspired oxygen
FPF: Fine particle fraction
HH: Heated humidifier
HME: Heat and moisture exchanger
kDa: Kilodaltons
MIC: Minimum inhibitory concentration
MV: Mechanical ventilation
MW: Molecular weight
NIV: Non- invasive ventilator
PaO2: Partial pressure of oxygen
PD: Pharmacodynamics
PEEP: Positive end-expiratory pressure
PK: Pharmacokinetics
pMDI: Pressurized metered dose inhaler
VHC: Valved holding chamber
VMN: Vibrating mesh nebulizer
